# Social Semiotics of Gangstalking Evidence Videos on YouTube: Multimodal Discourse Analysis of a Novel Persecutory Belief System

**DOI:** 10.2196/30311

**Published:** 2021-10-21

**Authors:** Andrew Lustig, Gavin Brookes, Daniel Hunt

**Affiliations:** 1 Department of Psychiatry Faculty of Medicine University of Toronto Toronto, ON Canada; 2 ESRC Centre for Corpus Approaches to Social Science Department of Linguistics and English Language Lancaster University Lancaster United Kingdom; 3 School of English Studies Faculty of Arts University of Nottingham Nottingham United Kingdom

**Keywords:** internet, discourse analysis, psychosis, delusion, semiotics, linguistics, computer-mediated communication, schizophrenia, eHealth, video, communication, YouTube, social media, discourse, mental health

## Abstract

**Background:**

Gangstalking refers to a novel persecutory belief system wherein sufferers believe that they are being followed, watched, and harassed by a vast network of people in their community who have been recruited as complicit perpetrators. They are frequently diagnosed as mentally ill, although they reject this formulation. Those affected by this belief system self-identify as targeted individuals (TIs). They seek to prove the veracity of their persecution and dispute the notion that they are mentally ill by posting videos online that purport to provide evidence of their claims.

**Objective:**

The objective of the study was to characterize the multimodal social semiotic practices used in gangstalking evidence videos.

**Methods:**

We assembled a group of 50 evidence videos posted on YouTube by self-identified TIs and performed a multimodal social semiotic discourse analysis using a grounded theory approach to data analysis.

**Results:**

TIs accomplished several social and interpersonal tasks in the videos. They constructed their own identity as subjects of persecution and refuted the notion that they suffered from mental illness. They also cultivated positive ambient affiliation with viewers of the videos but manifested hostility toward people who appeared in the videos. They made extensive use of multimodal deixis to generate salience and construe the gangstalking belief system. The act of filming itself was a source of conflict and served as a self-fulfilling prophecy; filming was undertaken to neutrally record hostility directed toward video bloggers (vloggers). However, the act of filming precipitated the very behaviors that they set out to document. Finally, the act of filming was also regarded as an act of resistance and empowerment by vloggers.

**Conclusions:**

These data provide insight into a novel persecutory belief system. Interpersonal concerns are important for people affected, and they construe others as either sympathetic or hostile. They create positive ambient affiliation with viewers. We found that vloggers use multimodal deixis to illustrate the salience of the belief system. The videos highlighted the Derridean concept of différance, wherein the meaning of polysemous signifiers is deferred without definitive resolution. This may be important in communicating with people and patients with persecutory belief systems. Clinicians may consider stepping away from the traditional true/false dichotomy endorsed by psychiatric classification systems and focus on the ambiguity in semiotic systems generally and in persecutory belief systems specifically.

## Introduction

Gangstalking refers to a persecutory belief system wherein those affected believe they are being followed, watched, and harassed by many people in their community who have been recruited into a network of complicit perpetrators [1]. In contrast to traditional forms of stalking that are usually organized by a single person [2], sufferers of gangstalking are unable to identify a responsible individual and experience it as a widely distributed and coordinated effort of co-conspirators.

Those affected gather in online communities to support each other and co-construct, develop, and contest the gangstalking belief system. The community members use a specialized lexicon to describe their experiences and to signal membership in the community of those affected. For example, the term “gangstalking” is used to describe the system of persecution, a “targeted individual (TI)” denotes the subject of the harassment, while those who participate in the intimidation are known as “perpetrators” or “perps” [3].

People affected by gangstalking report that the experience is extremely distressing. The campaign of harassment is frequently experienced as the accumulation of numerous otherwise innocuous acts, such as people clearing their throat, muttering under their breath, or giving lingering glances as they pass on the street. Because each of these acts may individually be passed off as unremarkable and mundane, TIs find it difficult to prove the existence of the harassment. When they come to clinical attention, they are frequently diagnosed with psychiatric illness and their belief systems are labeled as persecutory delusions [4]. However, TIs interpret such diagnoses as part of a gangstalking plan; by making them appear mentally ill, they are further discredited and stigmatized.

**Figure 1:**
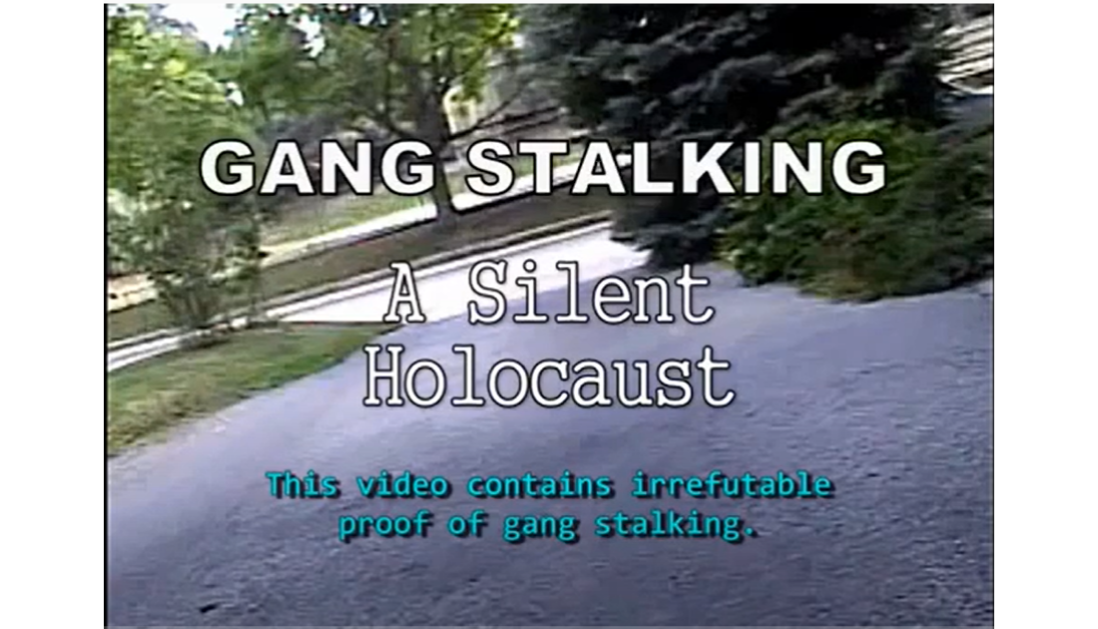
Gangstalking evidence video title screen.

Our previous work found that concerns about being believed and procuring and presenting proof of their systematic victimization are prime concerns of TIs in online fora [3]. For this study, we identified a genre of YouTube videos wherein TIs have posted videos to provide irrefutable evidence of their persecution and harassment. Figure 1 depicts a typical title screen of a video from this genre. YouTube provides a democratic and accessible medium where those affected can post videos to advance their point of view. In contrast to other social media platforms that are primarily text or image based, YouTube is a video-sharing platform. More than other media, video has an air of authority and legitimacy. Photography (and video) are often thought of as reproducing rather than representing reality [5], although, in reality, photographs and videos are often the product of a significant amount of arrangement and editing by their creators. These modes, in contrast, for example, to drawings or illustrations, possess a high degree of visual modality [6], presented as a seemingly “naturalistic, unmediated, uncoded representation of reality” [7]. Several culturally significant videos have demonstrated the evidentiary power of videographic evidence, such as the Zapruder video documenting the assassination of JFK and the Holliday video documenting the assault of Rodney King. Pop cultural artifacts, such as the British TV show *Caught on Camera*, highlight the role of the video camera as an apparently dispassionate and truthful observer of reality.

Previous studies have examined linguistic data in online communities regarding mental health concerns, including depression and anorexia [8], self-injury [9], the role of social media in construing subjectivity [10], and contested conditions, including diabulimia and gangstalking.

Previous studies have examined Google search activity in early psychosis [11] and the use of social networks in the diagnosis and treatment of psychotic disorders [12]. Previous research has also examined social media as a tool for family support [13] and as a diagnostic tool [14]. However, to date, there is no research examining multimodal semiotic content produced by people experiencing persecutory belief systems. This is the first study to examine social media content produced by people experiencing persecutory belief systems.

Table 1 summarizes recent research on computer-mediated communication by people with psychiatric illness.

**Table 1:** Recent research on computer-mediated communication by people with psychiatric illness.

Study	Year	Population	Finding
Kirschenbaum et al [11]	2020	Google search history prior to first admission for psychosis	People searched for information about symptoms prior to admission.
Birnbaum et al [14]	2020	Search history prior to diagnosis of psychiatric illness	Differences in search history were identified between healthy volunteers and people diagnosed with psychiatric illness.
Westermann et al [12]	2020	Internet-based intervention for people with psychosis	Psychotic symptom severity decreased in the internet-based intervention.
Barbetio et al [13]	2020	Systematic review of online interventions for families of patients with severe mental disorders	Perceived stress decreased in families.
Valentine et al [15]	2020	People’s experience of a social media–based intervention for first-episode psychosis	The intervention fostered connection and understanding.
Jakubowska et al [16]	2019	Systematic review of online social networking use among people with psychosis	People with psychosis appear to use online social networking frequently.

The objective of this study is to characterize the multimodal semiotic practices used in gangstalking evidence videos. We examine how these resources are deployed in order to construct the discourse of gangstalking, as well as how they are used to accomplish interpersonal tasks. To do this, we use a multimodal social semiotic theoretical framework to identify the semiotic resources used by TIs to construct and develop the gangstalking belief system in these evidence videos. In keeping with this framework, we identify social and interpersonal tasks accomplished by TIs in the videos, including constructing their own identity, creating distance with some groups, and fostering closeness and intimacy with other ones.

This analysis begins by describing and defining the genre of gangstalking evidence videos. We then describe how TIs use deictic strategies to create salience in the videos. First, we describe linguistic practices, and then we move on to visual strategies, including the gaze of the camera, intertitles, and text and image overlay. Next, we describe several interpersonal dynamics at work in the videos.

## Methods

### Collecting Videos

For this study, we assembled a corpus of videos posted on YouTube by self-identified TIs. For this, we used the YouTube Application Programming Interface (API) to search for videos. We used Python 3.0 to access the API and search the snippet object of videos. We searched for videos that contained the terms “gang stalking” or “gangstalking” AND “caught on video” or “caught on tape” or “proof” or “evidence” in the video title, description, or category. We sorted the search results by relevance using the YouTube API. We manually reviewed the search results to identify videos posted as proof or evidence of the gangstalking phenomenon. To be included in the analysis, a video had to be posted by a self-identified TI and purport to depict gangstalking activity. We identified other types of videos concerning gangstalking that were not germane to the analysis. These included first-person descriptive accounts of gangstalking, slideshow-type informational content about gangstalking, excerpts of news stories about gangstalking, original musical performances about gangstalking, and others. These videos were excluded from our analysis. We assembled a corpus of 50 videos meeting our inclusion criteria and achieved consensus by the authors regarding the suitability of the videos for inclusion.

### Multimodal Discourse Analysis

We then conducted a multimodal discourse analysis to identify the social semiotic resources mobilized by content creators posting YouTube videos presented as evidence of the gangstalking phenomenon.

To do this, we imported the videos into the NVivo software package (QSR International) [17], which allowed us to view the videos and annotate and code them. We applied a grounded theory approach to data analysis [18,19]. We viewed and transcribed the videos. We subsequently reviewed the transcripts and coded them. We grouped the codes into themes. We applied a qualitative multimodal discourse analysis to the video data, as described by Hansen and Machin [20].

We coded the videos and transcripts for paralinguistic and multimodal features of the videos, such as gestures, intertitles, text and image overlay, camera angles, and visual effects such as close-ups, time lapse, and slow motion. We considered linguistic and paralinguistic resources as well as the technologically mediated visual frame [21]. We captured these features in memos. We used a constant comparison approach, moving between the codes, memos, conceptual framework, and primary data until theoretical saturation was achieved.

All the data used for the analysis were posted in a public forum available to any internet user. Our analysis constitutes what Eysenbach and Till refer to as passive analysis [22].

The institutional research ethics board at the Centre for Addiction and Mental Health reviewed the proposed study design and opined that it did not require formal approval.

## Results

### Defining the Genre

Gangstalking evidence videos can be considered a subgenre of a video blog (vlog). A genre is a conventionalized form associated with a conventionalized purpose or occasion and is characterized by a schematic structure [23]. The videos contain several functional stages: an introductory section setting the stage, the body where the evidence is presented, and a coda that summarizes the evidence presented. To tell their story, authors rely on previously established genre conventions and tropes, in the process evidencing syntagmatic intertextuality, which has to do with how texts build on texts with which they are related sequentially [24].

As mentioned before, each video can be viewed as having three functional components (introduction, evidence, and coda), two of which are optional and one of which is essential to this genre.

#### Introduction

During the optional introductory component of the video, the TI addresses the viewer directly and orients them to the purpose and content of the video. As described later, TIs also use this section of the video to establish positive ambient affiliation with the viewer. Ambient affiliation is a concept proposed by Zappavigna [25] to describe the realization of social bonds in language and on the internet. Typical excerpts from the introductory section are as follows:

This is what a targeted individual experiences all day long.

All right, everyone. Looks like gangstalking is at hand. You guys wanted to see some video. Well. It's about to happen.

#### Evidence

This is the essential functional component of the video that presents the first-hand evidence of gangstalking. Much of the video is displayed in real time. However, two main temporal techniques are used to de-emphasize portions of the video deemed less significant or to highlight salient parts. Specifically, TIs use time lapse in parts that are deemed less important, while slow motion is used to mark particular moments in the video as important. We discuss these in more detail later.

#### Coda

During this optional component, the TI once again directly addresses the viewer, summarizes the contents of the video, and re-establishes affiliation with the viewer.

So there you go. There is a full-blown, orchestrated, uh, you know, I call it street theater–planned event, whatever. There it is broken down. All right, guys. Catch ya later. Bye.

In part, this generic structure derives its meaning from paradigmatic associations that the viewer establishes between the content of the video and other culturally available videos [26]. For instance, the use of the introduction and coda components to establish rapport with the viewing audience reflects similar practices in more conventional vlogging genres, while the use of introductory remarks to frame the subsequent evidence footage also reflects genres such as the *Caught on Camera* reality TV show or a nature documentary.

### Multimodal Deixis

A frequent and recurrent theme in the videos is that evidence of the gangstalking behaviors is obvious and self-evident; the actions of the people captured in the evidence videos are presented as incontrovertible evidence that they are gangstalkers. However, video creators also communicate that to pick up on the relevant cues, an observer must be oriented or initiated as to what cues to look for and be inducted into the group that is able to detect relevant signs. To bridge the gap between seemingly benign actions of people filmed by TIs and the gangstalking conspiracy, TIs use features of multimodal deixis to draw attention to salient features of the videos.

Deixis refers to linguistic features that encode information about the personal, spatial, and temporal situation in which they are used [27]. For example, successfully decoding and interpreting deictic expressions such as “here,” “this,” “that,” and “yesterday” requires some knowledge of the circumstances in which such expressions are used. In the case of video, the concept of deixis has been expanded beyond language to also include the visual mode. We consider how the video creators draw on both linguistic and visual deixis.

#### Linguistic Deixis

Most of the videos used some form of voice-over narration to describe their contents, put the depicted events in context, and create salience. This practice exemplifies discourse simultaneously being constructed by and constructing social reality; creators use deixis to draw attention to features and events that they observe and perceive as salient and, in so doing, construct the salience of these features for their viewers. In one video, a narrator claims:

There's a Honda Odyssey that always be following me, so.

The creator uses the deictic determiner *there* to direct the viewer’s attention to an otherwise unremarkable car in the video footage. The remainder of the sentence consists of a noun phrase (*a Honda Odyssey*), coupled with a relative clause that highlights the relevance or salience of this particular vehicle.

I see the neighbor who does not, uh, light up the front of his driveway with a security light. However, what he does do is he lights up the driveway of this house, which is 50 to 60 feet away. Now that’s called targeting. That’s called harassment.

In this example, the creator uses the deictic *that* as a demonstrative determiner to refer anaphorically to their prior description of the light. This gloss imbues the description with semiotic salience, highlighting the connotative significance of the scene. If this were absent, some uncertainty or confusion may exist regarding the relevance or significance of the light. These deictic phrases serve to definitively resolve this ambiguity.

Look at this guy. He is intentionally blocking me from moving.

The first sentence of this example serves to index the salient portion of the video. The second sentence then provides the connotative salience assigning malicious intent to the subject. In this way, seemingly normal elements in the video footage are verbally marked out for the viewer and then described as definitive evidence of gangstalking.

#### The Camera’s Gaze

**Figure 2:**
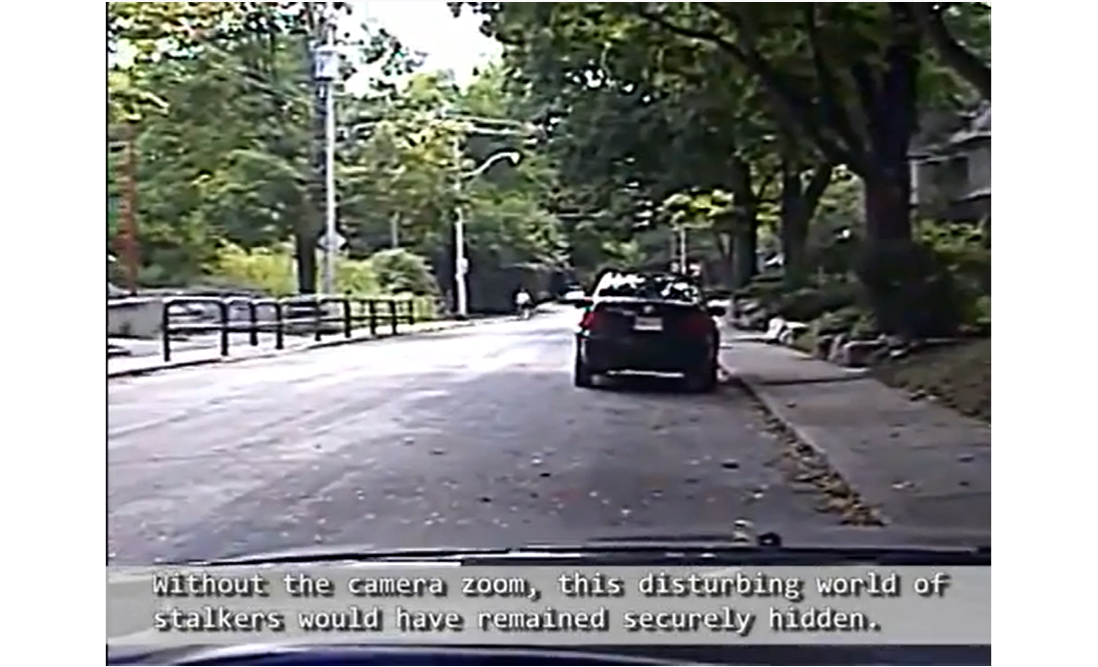
Still image illustrating the use of zoom.

The visual frame itself constitutes a type of deixis. By pointing the camera in a direction, at a particular scene, the image creator is effectively pointing at the scene and indicating that there is something relevant or noteworthy about it. In other words, image creators use the gaze of the camera to direct attention to salient content. The point-of-view (POV) shot is a shot in which the camera assumes the position of a subject to show us what that subject sees [28]. The POV shot also means that the TI’s and the audience’s gaze is conflated [21]. Within a shot, various other techniques are used to indicate salience: zoom, slow motion/time lapse, and repetition of salient sections of footage (Figure 2). The video bloggers (vloggers) in our data use zoom to highlight features and indicate their salience. This tactic is applied to the faces of perps. Vloggers used time lapse to speed up footage that was deemed of lesser importance, thereby de-emphasizing the salience of such segments. In contrast, slow motion was applied to highlight segments that were purported to be salient.

**Figure 3:**
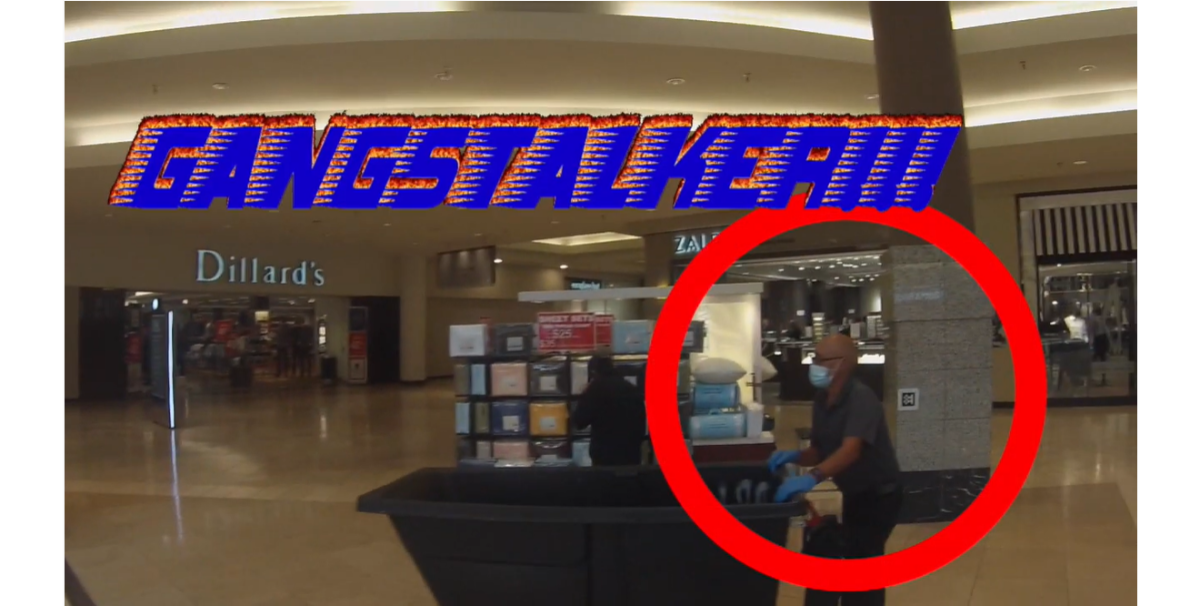
Image depicting visual deixis.

For example, one video is shot in a shopping mall and the evidentiary portion of the video depicts people walking around and shopping. A portion of the video depicts this in time lapse and then returns to normal speed to display Figure 3, which appears to be a caretaker pushing a garbage bin. The salience of the image is indicated by slowing the video from time lapse to real time and highlighting the salient feature with text overlay and a red circle.

#### Verb Transitivity

Systemic functional linguistics (SFL) uses the concept of transitivity to identify how speakers use language to describe their experience. It groups all processes, expressed by verbs, into six categories: material, behavioral, mental, existential, verbal, and relational [29]. Our analysis revealed that TIs frequently use material and behavioral processes to describe the activities of purported gangstalking.

In a video of a TI at a store, he says:

Literally look at ‘em all come over here.

Later in the same video:

Ha ha ha. It's so obvious. Absolute madness. Look at the whole store. Look at it. Barely anyone. Couple people here, and then wham, look at that. They just all came and flooded me like that, eh.

In this example, the TI uses the transitive material verb *flooded* with the direct object *me* to indicate that the malicious activity is being directed toward him. This lexical choice also invokes a water metaphor to equate the experience of being gangstalked to that of being flooded. Metaphor is pervasive in the language used to reify mental states and forms of distress [30]. This particular trope applies to gangstalking qualities that we may associate with flooding, such as it being excessive, uncontrollable, and overwhelming.

In contrast to material processes, which involve physical actions, behavioral processes exist at the border of material and mental processes and represent an external manifestation of cognitive processes [31]. Behavioral processes have only one participant, the behaver [32]. Additional information regarding the circumstances of the action is communicated by prepositional phrases. In this way, describing the actions of people in gangstalking evidence videos using behavioral processes at once allows video producers to mark their actions as ordinary *and* as evidence of more malicious thinking.

In this example, a vlogger documents his harassment at a gas station. Referring to a woman standing at the station, he says:

She's going to be constantly staring at me and no doubt running what happens in that store and who comes out of it and at what time.

By describing the woman’s behavior—“staring at me”—as constant, the video producer is able to represent this woman’s actions as suspicious, supporting the subsequent claim that she is “no doubt running what happens” in the store. Staring therefore becomes evidence of widespread, orchestrated harassment.

Behavioral processes such as this lie in the hinterland between thought and outward behavior. This is fertile ground for projection of the persecutory belief system. If the processes inferred were entirely mental, typically denoted by such verbs as *believe, hate*, or *know*, they would create no outward manifestation to document in the vlogs. In contrast, if the processes were entirely material, the observable behavior would not have the polysemous property that leaves it open to multiple interpretations. Occupying the middle ground between a physical and a mental process thus leaves these processes open to interpretation and hence open to classification as gangstalking.

#### Intertitles and Text Overlay

**Figure 4:**
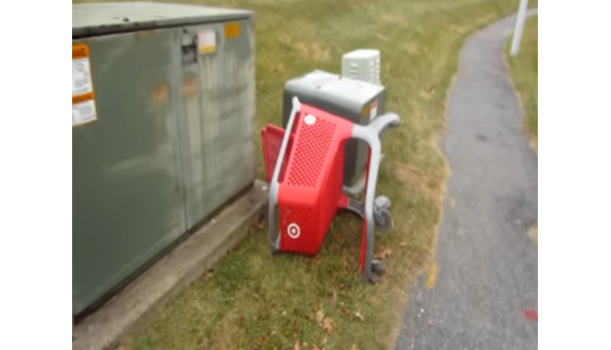
Unmarked image of a shopping cart.

**Figure 5:**
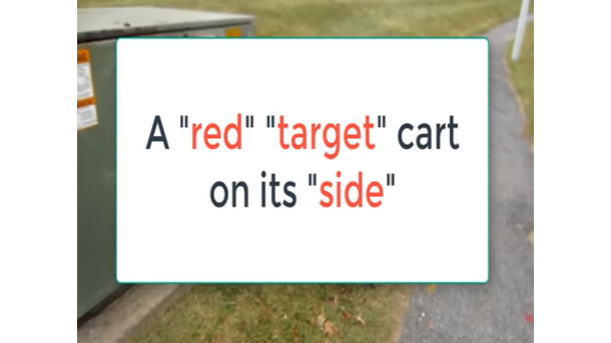
Title card suggests a deeper connotative meaning of the image.

**Figure 6:**
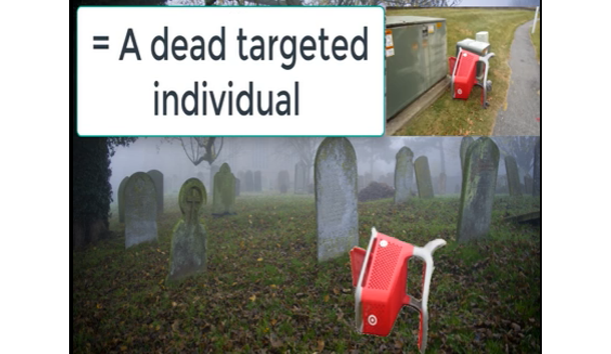
Third image in the series attempting to definitely define the significance of the image.

Vloggers make extensive use of text in apposition to video footage to create salience and construct the gangstalking narrative. Figure 4 depicts an urban scene with a shopping cart laying on its side in a park. This image is unremarkable on its own. The vlogger uses two follow-up images with text to construct this image as relevant to gangstalking. Figure 5 has the following text: *A “red” “target” on its “side”.* The use of quotation marks and red text highlights that these words are imbued with connotative meaning, although it remains unclear just what this meaning is. Figure 6, from the same video, attempts to definitively resolve the ambiguity with the text *= A dead targeted individual*. This gloss indicates that the apparently unremarkable scene is actually specifically about gangstalking and that the overturned cart represents a deceased TI. The connotation of death is intensified, and even rendered more explicit, by the image of a cemetery in the background onto which the shopping cart is overlayed.

**Figure 7:**
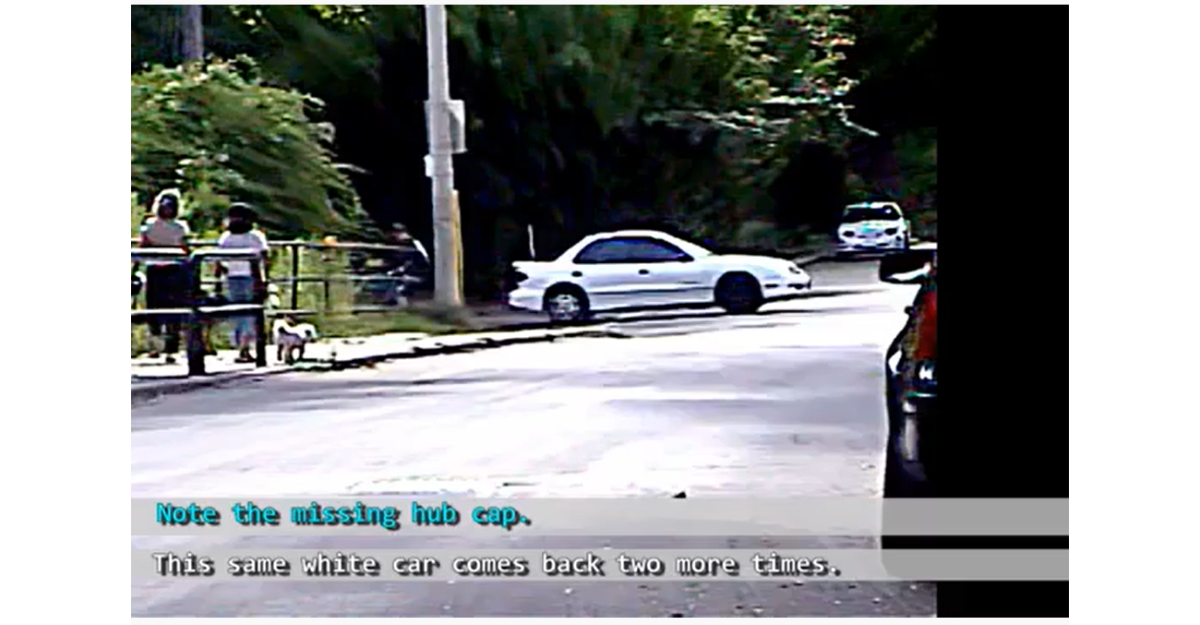
Combination of text and image assigns connotative significance.

In another example, an image depicts an outdoor urban neighborhood with people walking and cars being driven. As Figure 7 shows, a subtitle focuses attention on an otherwise unremarkable detail; a car depicted in the scene appears to be missing a hubcap. The subtitle urges viewers to *Note the missing hub cap*, thereby indexing this as salient. The text goes on to state *This same white car comes back two more times*. This combination of text and image allows the vlogger to tie together temporally disparate events and imbue them with salience, supporting the position that the driver of this car is engaged in gangstalking.

**Figure 8:**
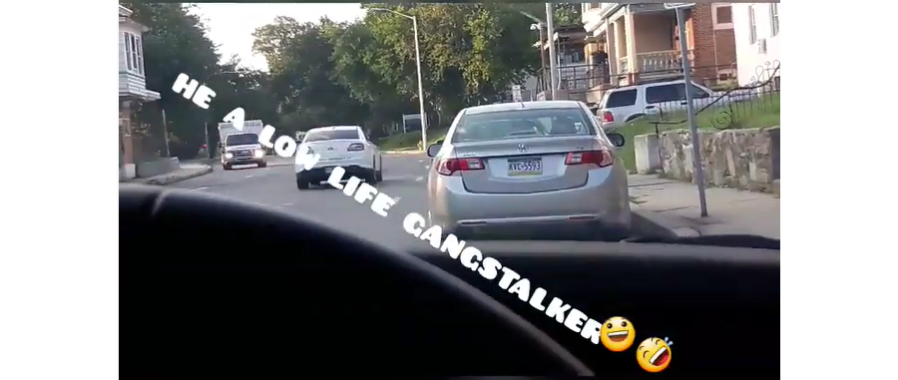
Use of text and emoji to construe salience of image.

A third example (Figure 8) depicts a street as viewed from the vantage point of the driver’s seat. The dashboard and steering wheel of the TI’s car frame the scene. The image is overlayed with the text *He a low life gangstalker*, followed by two emoji, one grinning and one laughing. Here, once again, the unindexed scene is unremarkable and does not itself suggest persecution or gangstalking. Rather, this interpretation is added by the overlayed text, which uses the deictic *He* to point out a participant in the scene and the phrase *a low life gangstalker* to functionalize the represented participant as engaged in gangstalking and to assign salience and significance to them.

### Ambient Affiliation With the Viewer

When posting videos on YouTube and other social media sites, a creator may direct them to a specific audience. More commonly, however, videos are merely “out there” for any other internet user to find and consume. They are meant for “anybody and everybody, or possibly nobody” [33]. This phenomenon has been called *context collapse*, referring to the numerous contrasting possible audiences and settings in which the vlogs may be viewed, including among audiences who may be skeptical of or hostile to the vloggers’ claims regarding gangstalking. TIs therefore use the videos to advance a discourse about the nature of gangstalking while simultaneously accomplishing interpersonal tasks relating to their audiences. They use one set of rhetorical techniques to generate affiliation with viewers of the videos and a contrasting set to promote disaffiliation and hostility with represented participants, which we term “representational disaffiliation.”

**Figure 9:**
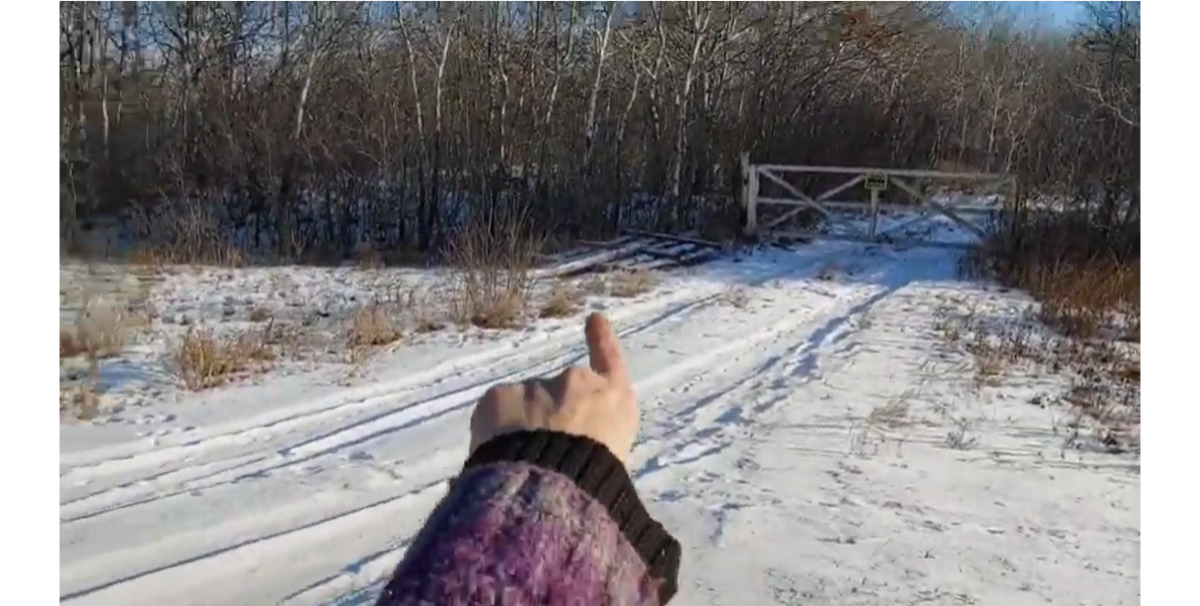
POV shot conflates the identity of the viewer and the targeted individual. POV: point of view.

TIs can choose between shots that depict them speaking to the camera and shots in which the camera assumes the position of a subject to show us what they see and to invite us to experience it contemporaneously with them. These compositional choices define the relationship between vlogger and viewer in a process called subjectification [10]. The main choice regarding subjectification is between positioning the viewer in an *as photographer* or a *with photographer* position. In our data, during the evidentiary portion of the videos, vloggers construct the former position and merge with the viewer, thereby achieving social affiliation. In some instances, a portion of the TI’s body is included in the shot, as in Figure 9.

Creators of the videos also use the introductory portion as an opportunity to affiliate with viewers. They gaze directly into the camera and arguably seek to establish a relationship with their viewers by looking directly at them. Such an image is known as a *demand* image [34] because the represented participants (in this case, the TIs themselves) engage the audience directly through their eye gaze and posture to request a relationship with them.

When addressing viewers directly, vloggers uniformly adopt a friendly, helpful, and explanatory tone as one might expect in an instructional video. TIs generally address viewers as equals; they do not use formal language or speak disrespectfully.

All right, everyone. Looks like gangstalking is at hand. You guys wanted to see some video. Well. It's about to happen.

In this example, the TI uses the politeness strategy [35] of recognizing the presumptive wants of their audience [27] to promote affiliation while also positioning themselves as part of a community of people interested in gangstalking.

What’s up YouTube? It’s your boy [redacted] coming at you with another one. You know what I’m sayin’?

In this instance, the TI adopts a friendly, conversational tone and informal forms of address that would not be out of place in more mainstream YouTube vlogs aimed at a young audience.

Yeah, guys, what's going on? Yeah, so something really crazy happened to me today.

Hey, guys. I've got some footage here of me filling up at a service station, and it shows just how the handlers orchestrate things.

Similarly, in the coda section of the vlogs, vloggers adopt a similar register with viewers:

Peace and love and light to all. I'm out.

Thank you. I'm [redacted], and I'm out in Oklahoma. September 15th, 2:05 pm. Have a good day.

But anyways, guys I'm gonna get off here for now. Um, enjoy your day. Unfortunately, it's rainy. Cloudy and rainy here today, but it's still a beautiful day, you guys. Stay happy. Stay peaceful. And yeah, just keep the hope going. Everything's gonna be good. Love you, guys. Bye.

### Representational Disaffiliation

In contrast to the affiliation construed with viewers, TIs use a different set of multimodal discursive strategies to construct others as being hostile and malicious and to increase interpersonal distance between themselves and the perps they claim are represented in the videos.

In contrast to the generally positive regard creators demonstrate toward viewers, they use a variety of discursive strategies to express negative affect—primarily anger and hostility—toward other people present in the videos. They also use compositional strategies to distance themselves from those represented as engaged in gangstalking.

**Figure 10:**
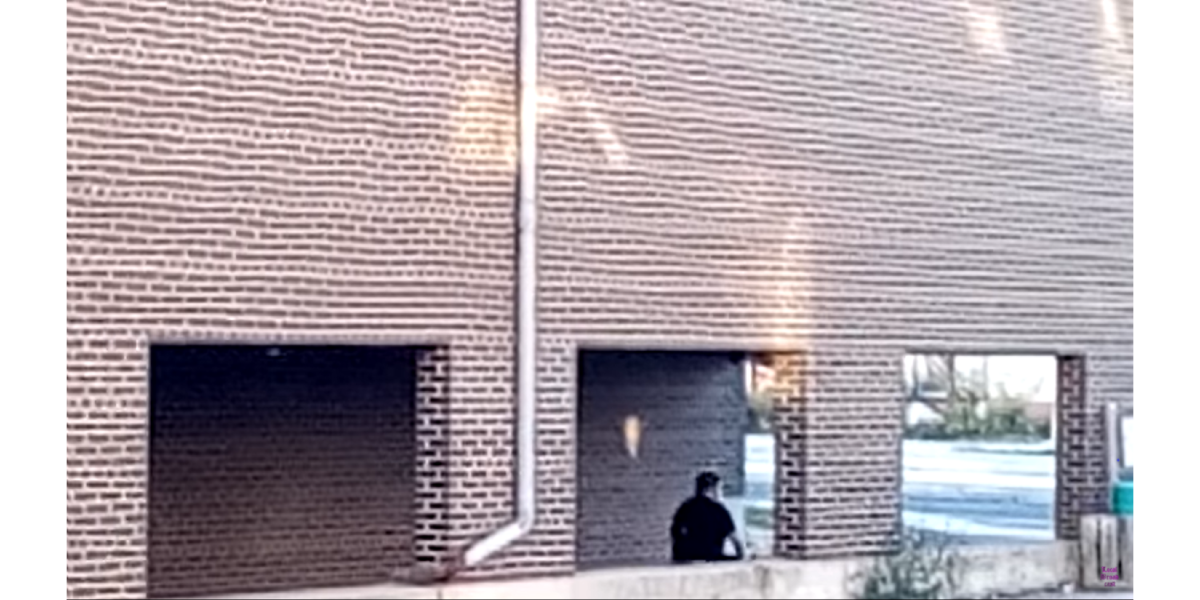
Long-distance shots create distance between perps and the vlogger/viewer dyad. vlogger: video blogger.

**Figure 11:**
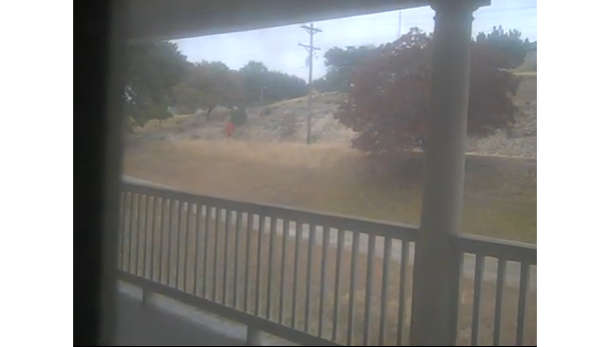
Use of framing disconnects perps from TIs. TI: targeted individual.

In the evidentiary portion of the vlogs, depictions of perps of gangstalking use long-distance shots, as shown in Figure 10. This generates interpersonal distance between perps on the one hand and the vloggers and viewers on the other [34]. As Figure 11 demonstrates, TIs also make extensive use of framing to disconnect themselves from the perps represented in the videos.

#### Uses of Impoliteness Strategies

TIs engage in a variety of face-threatening acts with others in their videos. “Face” is a sociological concept, originating in the work of Goffman [36], which broadly denotes individuals’ public self-image. Politeness strategies can be used to preserve but also to attack or harm another’s face [37]. The latter are known as face-threatening acts, and there is evidence of these in the data. In one video, a creator confronts a perp in a parking lot and says:

Why are you gangstalking? Why are you gangstalking me? Why are you gangstalking me? Why are two cars pulled up with their headlights facing me? Are you doing a psyop?

This utterance is face threatening in that it includes direct questioning but with no attempts at softening or redressing its direct, pointed nature. The formulation “Why are you gangstalking?” presupposes that the interlocutor is in fact engaged in gangstalking and leaves no room for a version of events in the way that the question “Are you gangstalking me?” would. Further, repeating the question several times in rapid succession lends a sense of urgency and even aggression to the interrogation.

In another video, a TI enters a Walmart store and confronts a group of people smoking cigarettes at the store entrance:

Aye, my god, you know there's, like, a cigarette place you can smoke over there? It's right there to the left. You feel me? So, like, you smoke a lot right here, that shit, like, that's fucked up. You know what I'm saying? But you could smoke right there, though.

In this example, the TI enacts impoliteness by calling out the behaviors of the interlocutors, who were apparently not known to him previously, and using expletives in pointedly criticizing their behavior [37]. He also uses message enforcers (*You feel me, You know what I’m saying*), which intensify the impoliteness of the utterance.

Immediately after disengaging from the group, the vlogger turns to the camera and says, “*That’s provoking. That’s provoking*,” to justify the hostility he demonstrated toward the smokers. He goes on to reiterate his high regard for viewers of the video and especially those who leave supportive comments:

I'm tired of this shit, boy. But I'm not gonna feed into it. Imma keep doing my thing. I appreciate you all up in my motherfucking comments, talking about some “Oh, don't feed into it.” But I've been ignoring this shit for too long.

This abrupt change of tone signals to viewers that although the TI is angry with those portrayed in the video, viewers are exempt from this and are in fact valued. Taken together, these strategies function to establish in- and out-groups around the vlogs. Although viewers are addressed using the language of social intimacy and as part of a shared community, perps who appear in the videos are explicitly confronted, interrogated, and sworn at. Despite engaging in such socially proscribed behavior, the way in which gangstalking vloggers explicitly justify their impoliteness toward perps also serves to present themselves as generally reasonable and polite; they mark their rudeness as an acceptable way to treat their purported harassers but also as a deviation from the otherwise genial register with which they address their intended audience. Nevertheless, such confrontational behavior creates a spectacle in the videos themselves, allowing the audience to vicariously experience direct altercations with apparent perps.

### Filming as an Act of Resistance

Throughout our data, and as indicated in the extant literature regarding gangstalking [1,3], TIs express that the persecutory system is so pervasive, persistent, and widely distributed that they feel powerless to effectively intervene or protect themselves. They note that law enforcement and other figures of authority are complicit, so the usual forms of redress are not available. The important caveat to this sense of futility and nihilistic outlook is that vloggers describe that perps are frightened of being recorded and of having their behaviors exposed publicly.

And it's to a point where I'm, like, OK. Cool. You all wanna be about that life, I be about it too. I'll record all your all ass . . . you try to provoke me, I'll put your ass on this camera.

Faces turned. They know that they're being filmed now. Look, they turn away. They shy away.

Tell you y'all. These damn demons won't stop. And I ain't gonna stop recording them.

By using the notion of perps being caught unawares and being unwillingly publicly exposed, the TIs can offset the power differential to some degree and partially restore a sense of agency. Creators frequently refer to perps as being frightened of exposure. The power of the videos, or more specifically the act of filming, to strike fear in perps is thus presented as helping TIs to redress the perceived power imbalance inherent in the persecutory belief system, wherein it is otherwise the perps who maliciously wield their collective power over TIs.

### Filming Perceived as an Aggressive Act

In some instances, community members appear unnerved by vloggers filming them, and the act of filming itself forms the basis of hostile interactions between vloggers and participants in the evidence videos.

In this instance, a vlogger confronts shoppers at a grocery store. He films them and accuses them of gangstalking him. He demands to see the manager, and when the manager arrives, the following exchange ensues, highlighting the act of filming as a source of conflict.

TI: Ah, here you are, mate. How are you? Good?

Manager: I'm good. How are you?

TI: Yeah, not bad. What's your name?

Manager: Why are you filming everyone?

TI: Oh, don't worry, man. I will . . . I won't post this on YouTube, I just want . . .

Manager: No, no. I'm just asking why you're filming everyone.

TI: I'm not filming people. I just wanna ask you a question.

Manager: No, no. You are filming, innit?

TI: No, no. Uh. No, no. Uh. Uhm. Can I ask you a quick question?

Manager: Can you stop that, please?

TI: Yeah, yeah. Of course.

TIs regard their filming as gathering evidence in an objective manner about activities that occur, regardless of whether they are being filmed. However, perps regard the process of filming itself as aggressive, hostile, and unnerving and respond with their own face-threatening acts by insisting that the filming cease. The act of filming, which is designed to capture hostility, appears to elicit the very phenomenon it attempts to document, thereby serving as something of a self-fulfilling prophecy.

## Discussion

### Summary of the Findings

Semiotics, the study of signs, defines a sign as composed of a signifier and a signified [38]. A sign is anything that can stand for something else. In semiotics, a *floating* or an *empty* *signifier* is one with a vague, highly variable, unspecifiable, or non-existent specifier [39]. The behaviors and interactions that form the subject of the gangstalking evidence videos analyzed in this study can be productively viewed as floating signifiers. They are polysemous in that different people may form distinct interpretations of the same observations. The scenes depicted appear to be unremarkable depictions of people going about their quotidian routines. The denotative meanings of these scenes are straightforward, but the connotative meanings are contested. The connotative significance of signs is used to signify the discursive content [40], in this case that gangstalking is real.

Our analysis revealed that the vloggers, who identify as individuals targeted by gangstalking, use a variety of multimodal strategies to indicate the salience of the acts depicted, thereby construing the gangstalking narrative. These included linguistic deictic features, paralinguistic features, and features operating through the visual frame.

Although the stated purpose of the videos is to document and disseminate evidence of the gangstalking phenomenon, vloggers also accomplish interpersonal tasks in the videos. On the one hand, they generate intersubjective ambient affiliation with viewers of the videos. On the other, they create hostility and reinforce animus with people depicted in the videos—a process we termed “representational disaffiliation.” This may serve to strengthen community building with viewers by projecting authenticity and intimacy [41]. People experiencing psychosis are likely to experience loneliness and social isolation at higher rates than the general population [42]. Our study is consistent with previous observations that people experiencing persecutory belief systems are more likely to perceive ambiguous social situations as hostile [43]. As predicted by interpersonal theory, this perceived hostility begets further hostility, leading to a positive feedback loop of ever-increasing hostility [44]. Pervasive hostility in the social milieu of TIs may frustrate the capacity to form strong, supportive interpersonal bonds in their offline lives. The need to have such bonds and to belong is a powerful, fundamental, and extremely pervasive motivation [45]. By forming affiliative bonds with hypothetical viewers of these videos, TIs may therefore offset the isolation induced by hostility and work toward meeting needs for belonging.

The videos use the rhetorical trope of synecdoche, where a part is used to stand in for the whole. Gangstalking is described as a widely distributed and pervasive system, but videos must be limited in scope in time and space due to practical constraints. The scenes depicted in the videos are meant to stand in for the pervasive nature of gangstalking. TIs are asking viewers of these videos to generalize the specific instances depicted.

The videos highlight the Derridean concept of différance. Derrida argued that “the signified concept is never present in and of itself, in a sufficient presence that would refer only to itself. Every concept is inscribed in a chain or in a system within which it refers to the other, to other concepts, by means of a systematic play of differences” [46]. In this study’s data, vloggers attempt to assign meaning through deixis. However, these efforts merely serve to defer meaning. When a vlogger points to a car and claims that it is being used for their persecution, a new question arises. Namely, what about that car signifies persecution? The vlogger may respond that it is some aspect of the car, such as its color, position, direction of travel, or the facial expression of the driver. However, this merely invites a new question: what is it about that attribute that signifies persecution? This cycle of deictic signifiers, each one pointing to the next, continues in an infinite chain and never arrives at its destination or definitively resolves the question.

### Clinical Implications

This observation may have important clinical ramifications. Traditionally, psychiatrists define delusions as fixed beliefs that are not amenable to change, considering conflicting evidence [47]. An alternative definition is that delusions are beliefs that are demonstrably untrue or not shared by others [48]. However, these and other definitions of delusions fall short, and arriving at a definitive definition may be impossible [49]. The prospect of a clinician definitively establishing the truth or falsity of a delusional belief system is often impractical or impossible. Often when a clinician states that a belief system is untrue or impossible, they are relying on their own beliefs, biases, and cultural referents. By shifting, instead, to a linguistic or semiotic understanding of delusions as belief systems that are unresolvable or that defer understanding ad infinitum, clinicians may sidestep the difficulties inherent in existing definitions. Ultimately, all users of semiotic systems—patients and clinicians alike—are subject to the same fundamental limits on communication and understanding inherent in language and all symbolic systems. Such a humbling realization may help to promote empathy and understanding and reduce stigma affecting people afflicted by persecutory belief systems.

### Conclusions

Our findings provide insight into a novel persecutory belief system. Interpersonal concerns are important for people affected, and they construe others as either sympathetic or hostile. They create a positive ambient affiliation with viewers. Vloggers use multimodal deixis to illustrate the salience of the belief system. Clinicians may consider stepping away from the traditional true/false dichotomy endorsed by psychiatric classification systems and focus on the ambiguity in semiotic systems generally and in persecutory belief systems specifically.

## Abbreviations

API: Application Programming Interface
POV: point of view
SFL: systemic functional linguistics
TI: targeted individual
vlog: video blog
vlogger: video blogger
